# How sleep quality and depressive symptoms shape safety behavior in rotating-shift nurses: Insights from mediation and network analyses

**DOI:** 10.1371/journal.pgph.0006008

**Published:** 2026-02-18

**Authors:** Huihan Zhao, Ping Huang, Huiqiao Huang, Shuyun Wang, Lin Lin, Zhaoquan Huang

**Affiliations:** 1 School of Information and Management, Guangxi Medical University, Nanning, Guangxi, China; 2 Department of Hematology, The First Affiliated Hospital of Guangxi Medical University, Nanning, Guangxi, China; 3 University Engineering Research Center of Digital Medicine and Healthcare, Guangxi Medical University, Nanning, Guangxi, China; 4 Key Laboratory of Hematology, Guangxi Medical University, Education Department of Guangxi Zhuang Autonomous Region, Nanning, Guangxi, China; 5 Department of Health Management and Division of Physical Examination, The First Affiliated Hospital of Guangxi Medical University, Nanning, Guangxi, China; 6 Department of Nursing, The Second Affiliated Hospital of Guangxi Medical University, Nanning, Guangxi, China; 7 Department of Pathology, The First Affiliated Hospital of Guangxi Medical University, Nanning, Guangxi, China; Universiti Kuala Lumpur Royal College of Medicine Perak, MALAYSIA

## Abstract

Safe behavior is critical for nurses to ensure patient safety, yet limited research has examined how physical and psychological health affect safety behavior in rotating-shift nurses. This study aimed to explore the underlying pathways and network connections among sleep quality, depressive symptoms, and safety behavior in rotating-shift nurses. A cross-sectional multicenter study focusing on rotating-shift nurses was conducted. Sleep quality, depressive symptoms, and nurses’ safety behavior were assessed using validated questionnaires. Correlation and mediation analyses were applied to explore the relationships and mediating pathways among sleep quality, depressive symptoms, and safety behavior. Network analysis was conducted to identify their internal associations and core symptoms. A total of 1,749 rotating-shift nurses were included in the study, with a relatively high safety behavior level (median score: 55). The prevalence of screen-positive poor sleep quality and moderate-to-severe depressive symptoms was 66.1% and 38.0%, respectively. Poorer sleep quality and depressive symptoms were associated with lower nurse safety behavior level (ρ = -0.167, ρ = -0.286, respectively). Moreover, the depressive symptoms mediated the effect of sleep quality on nurses’ safety behavior level. Network analysis further identified “*Insomnia, disturbed sleep*” of depressive symptoms, and “*sleep duration*” of sleep quality with higher centrality were bridge symptoms within the sleep-depression-safety behavior network. Rotating-shift nurses often experience poor sleep quality and depressive symptoms, which are associated with lower safety behavior level. Depressive symptoms may play a pivotal mediating role between sleep quality and safety behavior. Targeting key bridging symptoms – “*Insomnia, disturbed sleep*” and “*Sleep duration*” – may improve nurses’ sleep–depression–safety behavior network.

## 1. Introduction

Patient safety is central to any healthcare, but avoidable harm is rampant in all healthcare systems [1]. The “Global Patient Safety Report 2024”[1] reveals that over 10% of patients worldwide experience adverse events, and up to 12% of these result in permanent disability or death. Practicing safe behavior is a core ethical and professional standard for healthcare practitioners to ensure patient safety. However, their behaviors sometimes deviate from patient safety principles and clinical outcomes, occasionally, intentionally or unintentionally.

Safety behavior refers to the actions or behaviors exhibited by individuals in the workplace to promote the health and safety of workers, clients, the public, and the environment [2]. Nurses represent the largest segment of the healthcare workforce [3] and have the closest proximity to patients. Therefore, nurses’ safety compliance and participation behaviors may directly affect patient safety and outcomes in the healthcare system. However, recent studies have revealed that nurses’ safety behaviors remain suboptimal and are influenced by various factors, such as work environment, organizational support, safety knowledge and skill, motivation, culture, and professional identity [4–8]. Notably, nurses are required to provide 24-hour, uninterrupted healthcare services for patients. Shift work is unavoidable for nurses. Sustained exposure to shift-working demands may place considerable pressure on nurses and have profound implications for their sleep, mental health, and safety behavior.

Shift work leads to irregular sleep/wake patterns and chronic sleep deprivation, which can disrupt circadian rhythms [9]. Circadian rhythms regulate almost all cellular processes, and their disruption undoubtedly affects physiological and psychological health as well as behavior. Sleep problems in rotating-shift nurses have been well-documented [10–13]. Moreover, circadian disruption and poor sleep quality and quantity are key contributors to the long-term mental health effects among shift-working nurses [14]. Pandi-Perumal et al. have clarified that circadian rhythm alterations may underlie the mechanisms linking sleep disturbances and depression [15]. Similarly, Brown et al. argue that pronounced sleep disturbances are the fundamental causes of various psychological and behavioral problems [16]. Additionally, studies reported that nearly 20% of shift-working nurses suffer from shift work sleep disorder [17], 41.2% to 60.4% report poor sleep quality [18], 58.82% exhibited depressive symptoms, and 62.08% reported anxiety symptoms [19]. Therefore, sleep and mental health problems are highly prevalent and frequently coexist among shift-working nurses.

**Fig 1:**
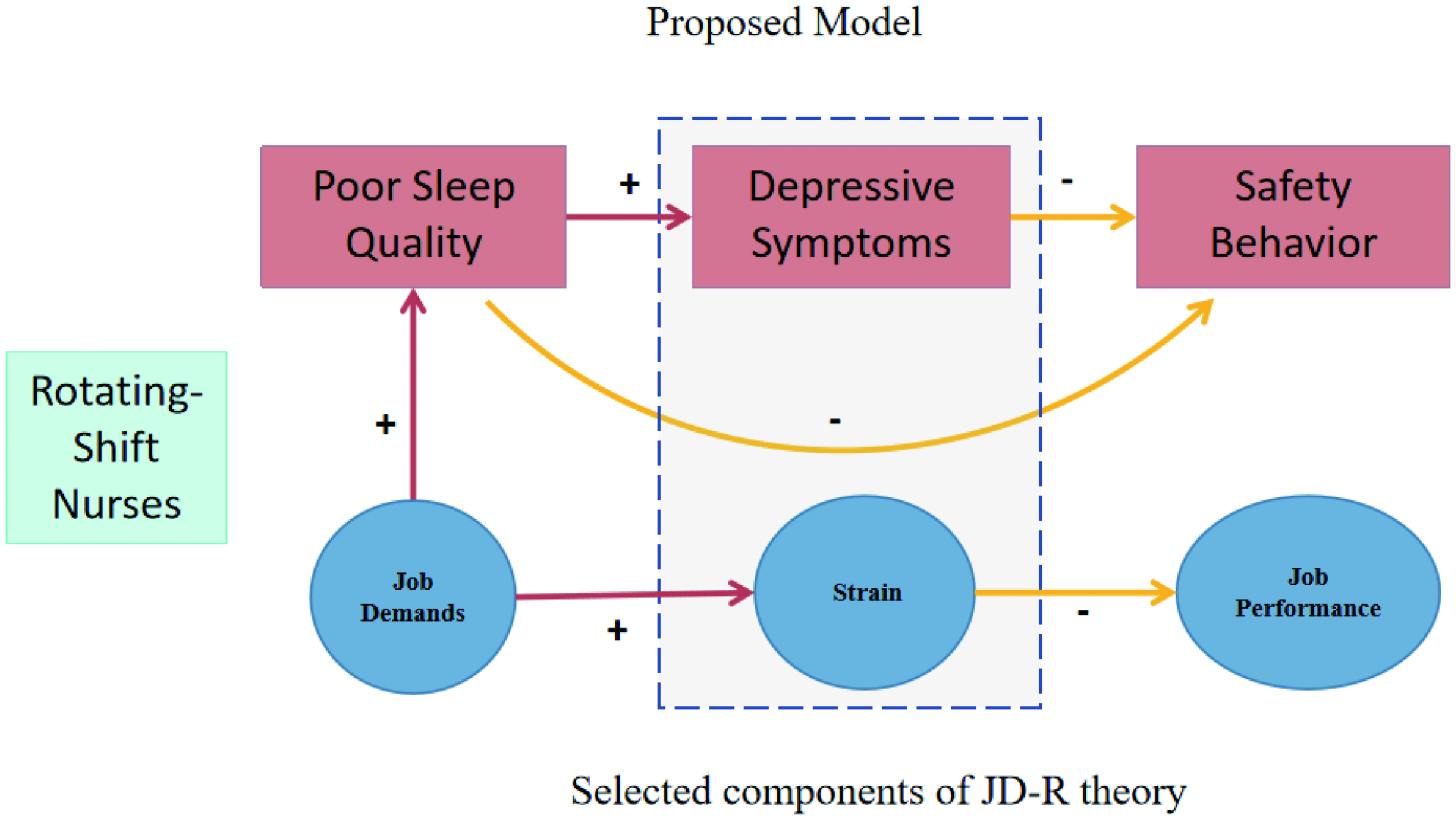
Theoretical framework and proposed model among sleep quality, depressive symptoms, and safety behavior in rotating-shift nurses. The upper section (red boxes) illustrates the key variables in this study: poor sleep quality, depressive symptoms, and safety behavior. The lower section (blue circles) highlights selected components of the Job Demands–Resources (JD-R) theory.

Poor sleep not only impairs nurses’ physical and mental health but also compromises their job performance and poses risks to patient safety [20–22]. The association between nurses’ mental health and patient safety has also been supported by large-scale evidence [23, 24]. Shift work disrupts circadian rhythms and sleep. This disruption can lead to cognitive impairments, such as attention variability and lapses, poorer working and short-term memory, worse executive functioning, and poor emotion regulation [25]. These cognitive effects may negatively affect rotating-shift nurses’ safety behaviors, job performance, and the overall quality of care. Moreover, animal behavior models also indicate that anxiety and major depressive disorder are associated with distinct avoidance and escape behavior patterns [26]. Other researches also show that depression and anxiety reduce safety motivation by impairing an individual’s ability to engage in necessary tasks or respond to external factors, thereby decreasing workers’ safety behaviors [27, 28]. Based on the Job Demands-Resources (JD-R) theory, sustained high job demands deplete physiological and psychological resources, potentially exacerbating psychological distress, subsequently disrupting daily functioning, reducing safety motivation, and impairing adherence to safety protocols [29]. In summary, sustained shift work demands could disrupt nurses’ circadian rhythms, causing chronic sleep deprivation, psychological resource depletion, and potentially impairing safety motivation, behavior, and performance. A theoretical framework and proposed model for this study is presented in Fig 1.

However, limited research has focused on rotating-shift nurses’ physical-psychological health and their safety behaviors. Given the complex interrelationships among sleep quality, depressive symptoms, and safety behavior, network analysis (NA) is particularly suitable for exploring their internal network structure and identifying core and bridge symptoms. According to network theory, NA has rapidly gained popularity in medicine as a computational approach to model the interconnections of symptoms [30, 31]. Rather than treating symptoms as isolated, the NA method considers potential causal relationships and assigns roles to symptoms based on their network position, connection strength, and overall connectivity [30]. The NA method represents symptoms as nodes and their relationships as edges, creating a complete visual graph of symptom interactions [30, 32]. By examining node centrality, core and bridge symptoms can be identified, highlighting the most influential symptoms in the network [30, 32]. The NA has become increasingly common in mental health research [32], but remains limited in sleep and behavioral sciences. Accordingly, we combined correlations, mediation analysis, and NA to explore the interrelationships, pathways, internal network structure, and bridge symptoms among sleep quality, depressive symptoms, and safety behavior in rotating-shift nurses.

## 2. Methods

### 2.1. Ethics statement

This study was approved by the Ethics Committee of the First Affiliated Hospital of Guangxi Medical University (Approval no. 2024-S652-01, approved on April 13, 2024). Informed consent was obtained from all participants.

### 2.2. Study design and participants

This cross-sectional, multicenter survey targeted rotating-shift nurses and was conducted in Guangxi, China. Eligibility criteria included individuals who held a valid registered nurse qualification, had been fully employed for at least six months, and engaged in frontline clinical nursing and rotating-shift work. Besides, participants were required to have experienced at least one evening shift (18:00–00:00) or night shift (00:00–08:00) in the past month, with a shift defined as a minimum of four consecutive working hours within the corresponding shift-working time period. Nurses undergoing clinical internships or training programs were excluded. Questionnaires were excluded if they had more than 5% missing data, multiple selections for single-choice items, contradictory answers to logically related questions, or responses inconsistent with the question content.

### 2.3. Data collection

The survey was conducted between May 15 and August 31, 2024, utilizing on-site and online survey methodologies. The on-site investigation was carried out by the primary researcher (H.Z.). The investigators obtained consent and scheduled recruitment by contacting nursing department directors or ward nursing managers at healthcare institutions. Eligible participants provided informed consent and then completed an anonymous paper-based questionnaire. The completed questionnaires were reviewed by the investigator for completeness before submission.

For the online survey, the survey link was created using the Online Questionnaire Star platform for distribution and data collection. Nursing department directors were contacted and invited to assist with participant recruitment, ensuring that all directors were familiar with the study’s inclusion and exclusion criteria. Then, the nursing department directors shared the survey link with eligible nurses through online channels. To minimize potential selection bias, all eligible rotating-shift nurses at each participating institution were invited to participate. Additionally, a unified set of guidelines was provided to participants, informing the study’s purpose, target population, significance, anonymity of the survey, voluntary participation, and privacy protection strategies. Participants were first required to electronically confirm their informed consent before proceeding with the questionnaire. Subsequently, they were instructed to answer all mandatory questions. Each participant could submit only one response. The primary investigator was responsible for addressing any questions or concerns that arose during the investigation.

### 2.4. Data collection instruments

The questionnaire consisted of four sections: general information and measurements of sleep quality, depressive symptoms, and nurses’ safety behavior levels. The general information section collected basic sociodemographic data (such as gender, age, ethnicity, institution, education level, professional title, and years of work experience), family-related information (such as marital status and number of children), and shift work-related information (such as shift patterns, number of night shifts in the past month, and number of rest days after night shifts). Sleep quality, depressive symptoms, and safety behavior levels were measured using validated instruments described below.

***Sleep quality*** The Pittsburgh Sleep Quality Index (PSQI) was developed by Buysse et al. [33] to assess sleep quality over the past month. The Chinese version of PSQI was validated by Tsai et al., demonstrating good reliability (α = 0.82–0.83) [34]. The PSQI consists of 19 items measuring seven specific components: subjective sleep quality (PSQI_C1), sleep latency (PSQI_C2), sleep duration (PSQI_C3), sleep efficiency (PSQI_C4), sleep disturbances (PSQI_C5), use of sleep medications (PSQI_C6), and daytime dysfunction (PSQI_C7). Each component is scored 0–3, yielding a total PSQI score ranging from 0 to 21, with higher scores indicating poorer sleep quality. A global PSQI score greater than 7 yields 98.3% diagnostic sensitivity and 90.2% diagnostic specificity for poor sleep quality [35]. In this study, the PSQI was used, with a global score >7 indicating poor sleep quality; Cronbach’s α = 0.734, showing acceptable reliability.

***Depressive symptoms*** The Patient Health Questionnaire-9 (PHQ-9), developed by Kroenke et al. [36], is a 9-item self-report tool that is widely used to screen depressive symptoms. The PHQ-9 has been validated in Chinese populations by Zhang et al. [37], demonstrating good internal consistency (Cronbach’s α = 0.854). Each item is scored 0 ~ 3, yielding a total of 0 ~ 27, with higher scores indicating more severe depressive symptoms. A cutoff score of ≥10 optimizes both sensitivity and specificity (both 0.85), effectively indicating the presence of moderate-to-severe depressive symptoms [38]. The PHQ-9 was employed in this study to assess depressive symptoms over the past month. A total PHQ-9 score ≥ 10 indicates screen-positive moderate-to-severe depressive symptoms in the study. The Cronbach’s α was 0.914, showing good reliability. Additionally, logically structured questions were included as a data quality check. Item 1 (“Little interest or pleasure in doing things”) was paired with its reverse-worded item (“Strong interest or pleasure in doing things”). Responses were classified as logically inconsistent and excluded if both items were answered as “not at all” or “almost every day”.

***Nurse safety behavior*** The Nurse Safety Behavior Questionnaire (NSBQ), initially developed by Shih et al. [39] and later translated into Chinese by Rong [40]. The NSBQ was used to evaluate healthcare workers’ behaviors in preventing or minimizing patient harm [39, 40]. It has been validated for use with Chinese healthcare professionals, demonstrating strong reliability and validity [41, 42]. The questionnaire consists of 12 items, with responses measured on a 5-point Likert scale ranging from “never” (1) to “always” (5). The questionnaire does not have a validated cutoff score for categorizing adequate or inadequate safety behavior. The overall scores range from 12 to 60, with higher scores indicating better safety behavior level. This questionnaire was used in the current study to measure nurses’ safety behaviors. The Cronbach’s α of the NSBQ scale was 0.931 in this study, indicating excellent reliability.

### 2.5. Data analysis

The paper-based survey data were entered into EpiData 3.1 software and subsequently exported to Excel format. The online survey data were directly exported in Excel format. Comparisons between the paper-based and online datasets showed no significant differences in PSQI, PHQ-9, or NSBQ scores (Mann–Whitney U test: p = 0.074, 0.443, and 0.708, respectively). The two datasets were then merged for analysis. All data were coded before analysis and accessed by the research team. All statistical analyses were conducted using various data analysis libraries, such as pandas, numpy, statsmodels, scipy.stats, seaborn, matplotlib.pyplot, networkx, and sklearn in Python. Missing data were minimal (10 values for total PSQI score, 0.57%) and were imputed using the median due to skewed distribution. Firstly, for non-normally distributed continuous variables, data were described using the median and interquartile range (IQR). Categorical variables were described as frequencies and percentages. Secondly, differences in PSQI, PHQ-9 and NSBQ scores between groups were analyzed using the Mann-Whitney U test or the Kruskal-Wallis H test.

#### 2.5.1. Correlation and mediation effect analysis.

Firstly, variables that showed significant differences in the univariate analysis were included as covariates, with categorical variables represented by dummy coding. To assess partial correlations among the PSQI, PHQ-9, and NSBQ scores after adjusting the covariates, we regressed each outcome on the covariates using generalized linear models (GLMs) and extracted the deviance residuals. This residual-based method enables flexible control of confounding under non-normal distributions and is widely used in psychological and symptom network research [43, 44]. Spearman’s rank correlation coefficients were then computed based on these residuals to estimate Spearman partial correlations.

Structural pathways were subsequently modeled, and mediation effects were analyzed using GLM-based regression in the statsmodels package. Bootstrap resampling with 1,000 iterations was applied to estimate confidence intervals for all regression coefficients.

#### 2.5.2. Network analysis.

Network analysis involves three key components: network description, network stability analysis, and network structure estimation [45]. In this study, network analysis was performed using the networkx package to explore partial correlations among the seven PSQI components, nine PHQ-9 items, and the total NSBQ score. To adjust for potential confounders (e.g., age, marital status, professional title, work experience, number of children, night shifts, and rest days), each variable was first regressed on these covariates using GLMs. Spearman partial correlations were then computed based on the deviance residuals and used to construct the network. Each item was represented as a node and the correlations between them as edges, with edge thickness reflecting correlation strength. A heatmap was generated to visualize the covariate-adjusted Spearman partial correlations, providing a complementary overview of association patterns.

Network and centrality graphs were used to visualize symptom connections, with edges included if absolute partial correlations exceeded 0.20. Network stability was assessed using the correlation stability (CS) coefficient, calculated through a case-dropping bootstrap method. For each bootstrap sample, we randomly remove 10% of the nodes from the original network, then recompute the degree centrality for the remaining nodes. The CS coefficient is computed as the average Pearson correlation between the degree centrality of the original network and the degree centrality of the bootstrap sample, repeated across 2500 case-dropping bootstrap iterations [43]. The value of the CS-coefficient above 0.5 is considered to have better stability [43]. The degree, betweenness, and closeness centrality were used for centrality measures. Sensitivity analysis was conducted by increasing the correlation threshold from 0.20 to 0.25 to assess the robustness of core and bridge nodes.

A p-value of less than 0.05 was considered statistically significant in all analyses. This study was reported in accordance with the STROBE guidelines.

## 3. Results

### 3.1. Participant characteristics and description

A total of 1,986 questionnaires were collected. After excluding 102 logically inconsistent responses and 135 with irrelevant answers, a total of 1,749 valid questionnaires were included. The effective response rate was 88.07%. The 1,749 respondents were from 63 medical institutions across 12 cities in Guangxi, China. Of these, 270 (15.4%) were from county-level hospitals, and 1,479 (84.6%) were from urban hospitals. The median PSQI score was 9 (IQR: 4), with 1,156 (66.1%, 1,156/1,749) respondents meeting the screening threshold for poor sleep quality (PSQI score > 7). The median PHQ-9 score was 9 (IQR: 5), with 664 (38.0%, 664/1749) respondents meeting the screening threshold for moderate-to-severe depressive symptoms (PHQ-9 ≥ 10). The median NSBQ score was 55 (IQR: 9).

Harman’s single-factor test was conducted to examine common method bias. The first principal component explained 30.2% of the variance, which was below the critical threshold of 40% [46]. This result indicates that common method bias was not a serious concern in this study. Nurses with marital status of divorce or those with junior/ intermediate professional titles had poorer sleep quality, more severe depressive symptoms, and lower safety behavior level (p < 0.05). Additionally, respondents aged between 20 and 30 years, with ≤5 years of work experience, and ≥3 children, showed a lower safety behavior level (p < 0.05). The number of night shifts and rest days after night shifts in the past month significantly affected their sleep quality and depressive symptoms (p < 0.05), but no significant difference was found in safety behavior level (p > 0.05). Detailed demographic information and characteristics are provided in Table 1.

**Table 1:** Sociodemographic characteristics of the participants (n = 1749).

Items	N (%)	PSQI M (IQR)	U or H Statistic/p value	PHQ-9 M (IQR)	U or H Statistic/p value	NSBQ M (IQR)	U or H Statistic/p value
***Demographics characteristics***							
**Gender**							
Male	101(5.8)	8(5)	90490/0.139	9(6)	78388/0.324	53(10)	88827/0.254
Female	1648(94.2)	9(4)		9(5)		55(9)	
**Age range**							
20 ≤ years<30	655(37.4)	9(4)	6.177/0.103	9(6)	**8.887/0.031**	54(10)	**29.140/ < 0.001**
30 ≤ years<40	882(50.4)	9(5)		9(5)		55(8)	
40 ≤ years<50	181(10.3)	8(5)		8(4)		57(7)	
years≥50	31(1.8)	9(6.5)		8(6)		57(7)	
**Ethnicity**							
Han	1069(61.1)	9(5)	3.328/0.190	9(6)	2.021/0.364	55(9)	5.104/0.078
Zhuang minority	590(33.7)	9(4)		9(5)		55(9)	
Other	90(5.1)	9(6)		8(6)		56(7)	
**Education level**							
Vocational High School	16(0.9)	9(5)	8.639/0.035	9(5)	3.817/0.282	55(10)	2.499/0.475
Associate degree	313(17.9)	9(4)		9(5)		55(8)	
Bachelor’s Degree	1399(80.0)	9(5)		8(3)		53(8)	
Master’s Degree or Higher	21(1.2)	12.5(5)		11(8)		52(9.75)	

***Work characteristics***							
**Hospital level**							
Urban hospital	1479(84.6)	9(4)	187224/0.102	9(5)	205414.5/0.449	55(9)	202354/0.724
County level hospital	270(15.4)	9(5)		9(4)		55.5(10)	
**Work experience**							
≤ 5 years	481(27.5)	9(4)	4.132/0.388	9(6)	**14.557/0.006**	53(10)	**27.926/ < 0.001**
5 < years≤10	529(30.2)	9(4)		9(6)		55(9)	
10 < years≤ 15	446(25.5)	9(4)		8.5(5)		55(7)	
15 < years ≤20	190(10.9)	9(5)		9(5)		55(7.75)	
Years＞20	103(5.9)	8(5.5)		8(3.5)		57(6.5)	
**Professional title**							
Junior and below	855(48.9)	9(4)	**6.284/0.043**	9(6)	**7.897/0.019**	54(10)	**20.567/ < 0.001**
Intermediate grade	817(46.7)	9(5)		9(5)		55(8)	
Senior	77(4.4)	8(5)		8(4)		57(6)	
**Shift pattern**							
DN	605(34.6)	9(5)	0.882/0.643	9(5)	0.199/0.905	55(8)	2.090/0.352
APN	952(54.4)	8(4)		9(6)		55(10)	
Other	192(11.0)	9(4)		9(6)		55(8)	
**Number of evening and night shifts in the past month**							
1	27(1.5)	9(5)	**33.986/ < 0.001**	9(4)	**13.245/0.010**	55(9)	2.216/0.696
2	96(5.5)	9(4)		9(6)		55(9)	
3 ~ 4	679(38.8)	10(5)		9(6)		55(8)	
5 ~ 8	612(35.0)	8(5)		9(4.25)		55(9)	
≥ 9	335(19.2)	7(4)		8(7)		56(7.5)	
**Off days after night shifts**							
1 ≤ days≤2	1470(84.0)	9(5)	**227494/0.004**	9(5)	**226358/0.006**	55(9)	209819/0.538
2 < years ≤3	279(16.0)	8(5)		8(5)		54(8)	
***Family characteristics***							
**Marital status**							
Married	1140(65.2)	9(4)	**8.910/0.030**	9(6)	**11.283/0.010**	54(10)	**13.545/0.004**
Unmarried	582(33.3)	9(4)		9(5)		55(8)	
Divorced	26(1.5)	10.5(4)		10(7.25)		53(9.75)	
Other	1(0.1)	4		1		58	
**Number of children**							
0	733(41.9)	9(4)	4.603/0.203	9(6)	7.666/0.053	54(10)	**19.875/ < 0.001**
1	463(26.5)	9(5)		9(4)		56(7)	
2	522(29.8)	9(4)		9(6)		55(8)	
≥ 3	31(1.8)	9(5)		9(5.5)		53(9)	

Notes: PSQI, Pittsburgh Sleep Quality Index (Sleep quality); PHQ-9, Patient Health Questionnaire-9 (Depressive symptoms); NSBQ, Nurse Safety Behavior Questionnaire (Safety Behavior); M, Median; IQR, Interquartile range; DN, Day-Night shift; APN, Day (AM)-Evening (PM)-Night (N) shift; Statistical values and p-values indicating significant differences between groups are bolded.

### 3.2. Correlations between the sleep quality, depressive symptoms, and safety behavior level

After adjusting for potential confounders, including age range, marital status, work experience, professional title, number of children, number of night shifts per month, and off days after night shifts, Spearman partial correlation analysis revealed significant pairwise correlations among the sleep quality, depressive symptoms, and safety behavior level. Poorer sleep quality (ρ = -0.167, p < 0.001) and depressive symptoms (ρ = -0.286, p < 0.001) were negatively correlated with nurses’ safety behavior level. In contrast, poor sleep quality was positively correlated with depressive symptoms (ρ = 0.531, p < 0.001).

### 3.3. Mediation effect

After adjusting for potential confounders, the direct effect of sleep quality on safety behavior level was not statistically significant (coef. = −0.031, p = 0.604). Mediation analysis revealed that this relationship was indirectly mediated by depressive symptoms, with an indirect effect of coef. = −0.221 and a total effect of coef. = −0.253. Detailed pathway effects are presented in Table 2. The complete estimates of direct, indirect (via PHQ-9 score), and total effects of sleep quality (PSQI score) and covariates on safety behavior level are provided in S1 Table.

**Table 2:** Mediation of depressive symptoms in the association between sleep quality and patient safety behavior among rotating-shift nurses.

Path	Coefficient [95% CI]	Z Statistic	p-value
***PSQI score* → *PHQ-9 score* → *NSBQ score (*** *Total effect* **)**	-0.253 [-0.362, -0.163]		
PSQI score → NSBQ score *(Direct effect)*	-0.031[-0.150, 0.087]	-0.518	0.604
PSQI score → PHQ-9 score →NSBQ score *(Indirect effect)*	-0.221[-0.284, -0.161]		
PSQI score →PHQ-9 score	0.807[0.748, 0.866]	26.773	<0.001
PHQ-9 score →NSBQ score	-0.274[-0.354, -0.195]	-6.784	<0.001

Notes: PSQI, Pittsburgh Sleep Quality Index (Sleep quality); PHQ-9, Patient Health Questionnaire-9 (Depressive symptoms); NSBQ, Nurse Safety Behavior Questionnaire (Safety Behavior level); CI, Confidence Interval.

### 3.4. Network structures

#### 3.4.1. Overall network structure.

**Fig 2:**
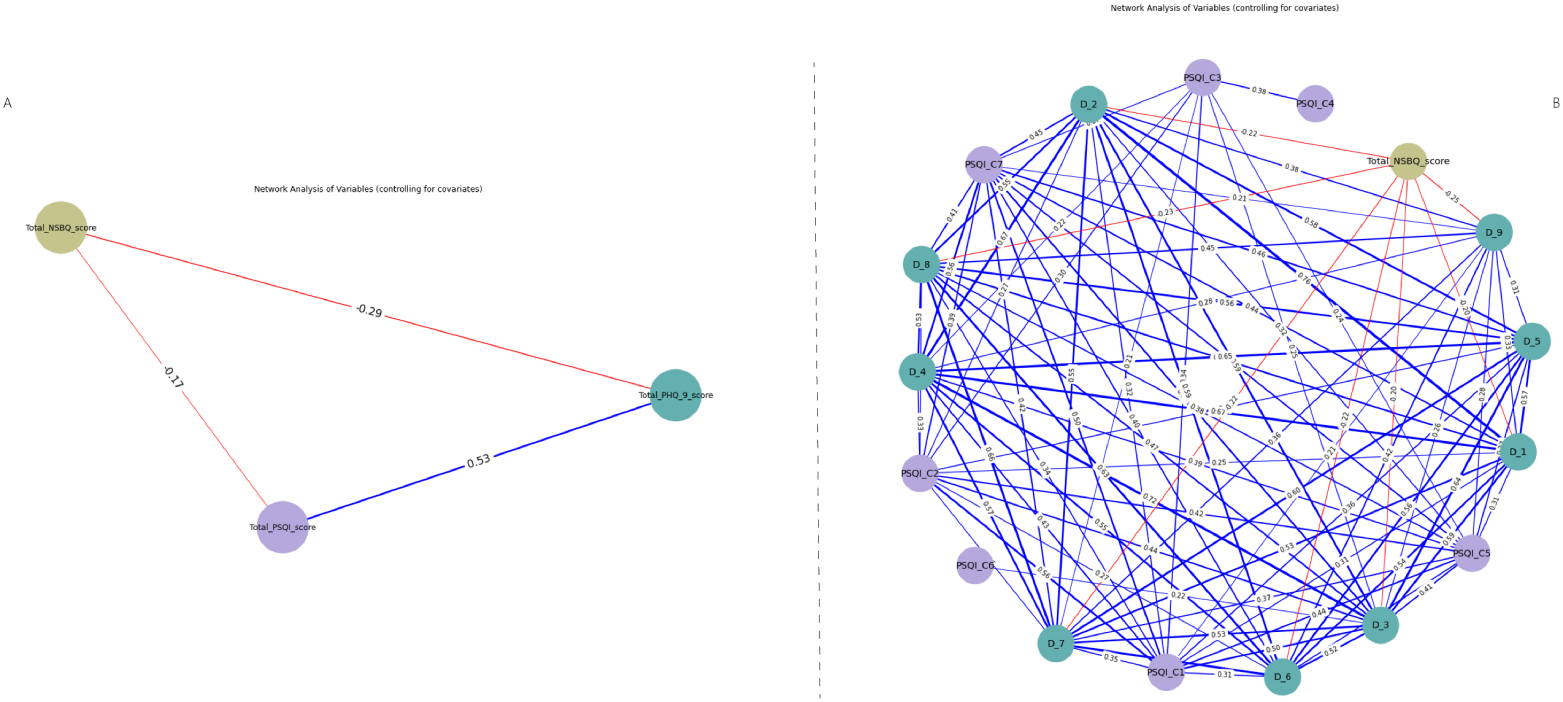
Network structure of sleep quality, depressive symptoms, and safety behavior in rotating-shift nurses. Fig 1 A shows the overall network connections among sleep quality, depressive symptoms, and patient safety behavior. Fig 1 B presents the internal network connection between sleep quality components, depression items and total safety behavior scores. Note: In this network, each node represents an item (PSQI_C1-PSQI_C7 for PSQI components, D_1-D_9 for PHQ_9 items), and the relationships between them as edges. Blue edges indicated positive correlations, while red edges represented negative ones, with edge thickness reflecting correlation strength. Node colors indicate variable type: purple for PSQI components, green for PHQ-9 items, and yellow for safety behavior.

The network analysis of the total PSQI, PHQ-9, and NSBQ scores revealed that the direct correlation between sleep quality and safety behavior level was weaker, while the associations between sleep quality and depressive symptoms, as well as depressive symptoms and safety behavior level, were stronger (Fig 2A).

#### 3.4.2. Internal network structure.

**Fig 3:**
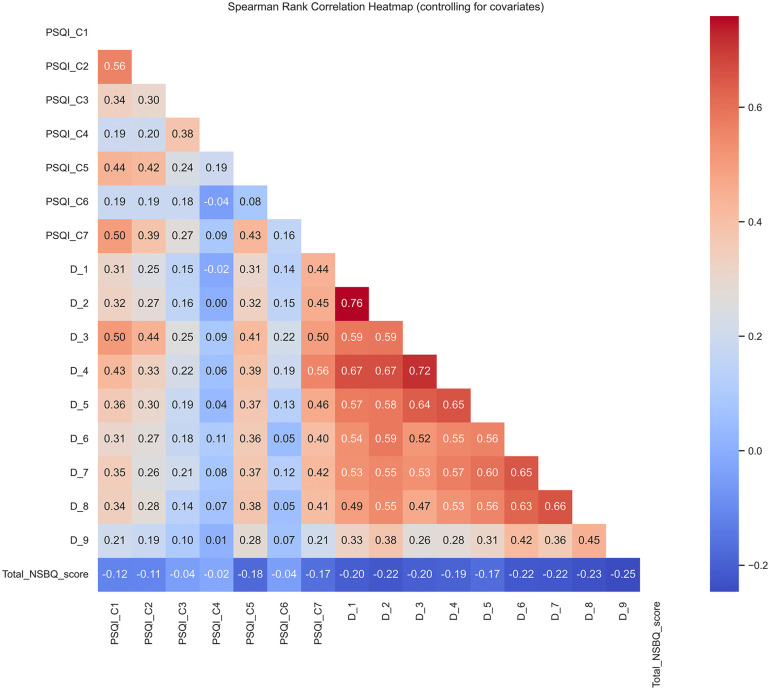
Heatmap of the correlation between sleep quality components, depressive items and total safety behavior scores. Partial Spearman correlations are shown among sleep quality components (PSQI C1–C7), depressive symptoms (PHQ-9 D1–D9), and the total safety behavior score (Total NSBQ score), controlling for age, marital status, professional title, work experience, number of children, night shifts, and rest days. Only the lower triangle of the correlation matrix is displayed. Color intensity reflects the strength of the correlations, with darker shades indicating stronger associations. The depressive symptoms exhibited stronger associations with the total safety behavior level (ρₚ range: -0.25 to -0.17) compared to the sleep quality components (ρₚ range: -0.17 to -0.02).

The network connections among PSQI components, PHQ-9 items, and the total NSBQ score are presented in Fig 2B. The results indicate that nodes of depressive symptoms exhibited stronger associations with the total safety behavior level (ρₚ range: -0.25 to -0.17) compared to the nodes of sleep quality (ρₚ range: -0.17 to -0.02). The Spearman partial correlation coefficients between nodes are presented in the heatmap (see Fig 3). Detailed correlation coefficients and their 95% confidence intervals for all node pairs are available in S2 Table. The network’s density was 0.684, indicating strong connectivity, and its diameter was 3, meaning the longest shortest path between any two nodes was 3 edges. The average path length of 1.38 suggests efficient information flow within the network. The average clustering coefficient was 0.79, indicating substantial local clustering among nodes. The variance in node degrees was 17.81, reflecting significant variability in the number of connections across nodes. The CS coefficient for the degree centrality was 0.997, indicating high stability across different bootstrap samples.

#### 3.4.3. Node centrality and bridge symptoms in the internal network structure.

**Fig 4:**
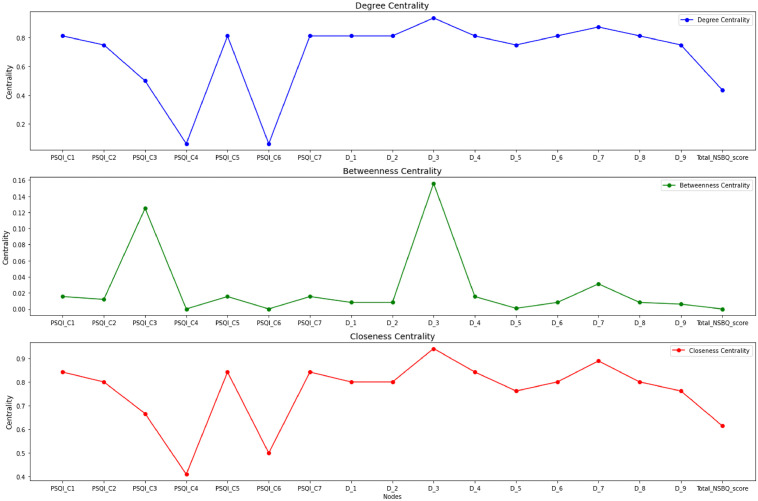
The centrality diagrams of the sleep quality-depressive symptom-safety behaviors networks in rotating-shift nurses. Centrality reflects the relative prominence and importance of a node within the network. In this network, each node represents an item (PSQI_C1-PSQI_C7 for PSQI components, D_1-D_9 for PHQ_9 items). Total NSBQ score indicates the level of nurses’ safety behavior. D_3 and D_7 had the highest degree centrality and closeness centrality. Additionally, D_3, PSQI_C3, and D_7 exhibited the highest betweenness centrality, indicating that they served as bridge symptoms within the network.

The centrality diagrams of each node are presented in Fig 4. The node centrality metrics are given in S3 Table. Notably, D_3 (“*Insomnia, disturbed sleep, or excessive sleep*”) and D_7 (“*Trouble concentrating on things, such as reading the newspaper or watching television*”) had the highest degree centrality (15 and 14, respectively) and closeness centrality (0.941 and 0.889, respectively), identifying them as the potential core symptoms in the network. Additionally, D_3, PSQI_C3 (“*Sleep duration*”), and D_7 exhibited the highest betweenness centrality (0.156, 0.125, and 0.031, respectively), indicating their potential roles as bridge symptoms in the network. Furthermore, 70.6% of the nodes had clustering coefficients above 0.90, suggesting a high degree of local interconnectivity in the network.

Sensitivity analysis showed that, when a stricter correlation threshold (|r| ≥ 0.25) was applied, D_3 consistently retained the highest degree and closeness centrality, confirming its stable role as the core symptom. PSQI_C3 and D_3 also maintained the highest betweenness centrality, supporting their robust role as bridge symptoms. In contrast, D_7’s central role was attenuated under this specification, indicating that its core and bridging functions were sensitive to threshold changes.

## 4. Discussion

Although determinants of employee safety behavior have been studied across various industries [47, 48], research on rotating-shift nurses, particularly focusing on the physiological–psychological–behavioral pathway, has been scarce. This study revealed the relationship, pathways, and network between sleep quality, depressive symptoms, and safety behavior among rotating-shift nurses in Guangxi, China. Our findings contribute to a deeper understanding of the sleep quality-safety behavior link, offering valuable insights for developing targeted interventions to improve both rotating-shift nurses’ health and patient safety.

Rotating-shift nurses experience a high burden of poor sleep quality and moderate-to-severe depressive symptoms, with screen-positive prevalence rates of 66.1% and 38.0%, respectively, based on validated self-report questionnaires rather than clinical diagnoses. These findings align with studies in healthcare workers worldwide. For instance, a study from France found that 29.8% of healthcare workers experienced depression, and 64.5% reported poor sleep quality [49]. A cross-sectional survey from Japan reported that 41.2% to 60.4% of shift-working nurses and midwives experienced poor sleep quality [18]. A multi-center cross-sectional online survey from China found that 58.82% and 62.08% of shift nurses experienced depressive symptoms and anxiety, respectively [19]. Although, the differences in prevalence rates across studies persist and may be attributed to various regional and organizational differences, such as economic pressures, workplace conditions, work culture, shift work schedules, and mental health support. All these studies consistently highlight that shift-working nurses face significant sleep and mental health challenges.

Shift work often requires being awake at night and sleeping during the day, which leads to shorter and less restorative sleep due to circadian misalignment and social constraints from family and work responsibilities. Therefore, most shift workers report reduced sleep quality and duration [50]. Additionally, significant sleep disturbances are a key foundation for shift work-related psychological and behavioral issues [16] and are also reported as an early indicator of depression [51]. Therefore, the prevalence of screen-positive poor sleep quality and depressive symptoms is high among rotating-shift nurses. This finding highlights the urgent need to optimize shift designs and implement targeted interventions to support sleep and mental health in this population.

Despite experiencing significant sleep disturbances and depressive symptoms, the rotating-shift nurses can maintain a relatively high level of safety behaviors, with a median NSBQ score of 55. This finding is similar to a study of tumor-specialized nurses in China, which reported a total safety behavior score of 55.45 ± 6.879 [41], and another study focusing on psychiatric nurses, which reported scores of 47.98 ± 7.45 [52]. These high levels of safety behaviors among shift-working nurses may be related to their rigorous professional education and training. Besides, we further found nurses aged between 20 and 30 years, with a marital status of divorce, junior/ intermediate professional titles, ≤ 5 years of work experience, and ≥3 children, showed lower patient safety behavior levels. These findings align with previous reports indicating that less experienced nurses and younger age groups tend to have lower safety behavior levels [41, 52]. Researchers suggested that this may be attributed to both individual and systemic factors, such as healthcare personnel’s knowledge and attitudes, nurse collaboration, information systems, standardized care protocols, and patient participation [53]. We speculate that these disparities may also be related to insufficient risk perception, lack of safety training, limited work experience, and family-to-work conflict.

Notably, as most existing studies rely on self-reported measures, they may intentionally underreport unsafe practices or be unaware of their unsafe behaviors. Therefore, rotating-shift nurses’ safety behavior levels might be overestimated, potentially due to social desirability bias in the nursing profession, where safety compliance is widely regarded as a professional and ethical standard. The high median NSBQ score, close to the upper limit of the scale, suggests a potential ceiling effect that may have reduced score variability and attenuated observed associations between sleep quality, psychological status and safety behavior levels. Therefore, field-based observational studies and more objective assessment methods are needed to more accurately capture safety behaviors in healthcare settings.

We observed that shift nurses’ poorer sleep quality was positively correlated with depression level (ρ = 0.531), while poorer sleep quality (ρ = -0.167) and depressive symptoms (ρ = -0.286) were negatively correlated with their safety behavior level. Sleep problems and depressive symptoms are common among shift workers [9, 10]. The relationship between sleep quality and mental health has also been documented in previous studies [54–56]. Circadian rhythm alterations may underlie the mechanisms linking sleep disturbances and depression [15]. However, to date, no study has explored the pathways through which sleep quality and mental health influence individual safety behaviors. Our findings further reveal that poorer sleep quality may influences rotating-shift nurses’ safety behaviors through indirect effects of depressive symptoms (coef. = -0.221) more than direct effects (coef. = -0.031). This can be better understood from the perspective of behavioral psychology. Long-term sleep deprivation due to shift work triggers a psychological chain reaction that can ultimately affect safety behavior motivation, safety compliance, and participation behaviors.

In rotating-shift nurses, poor sleep quality may not directly influence safety behavior motivation or performance, but may exert an effect indirectly through psychological states. The JD-R theory provides a robust framework for understanding these dynamics. The JD-R model has been widely applied in nursing contexts; for example, recent studies have extensively explored the relationships among work environment, job demands, job resources, burnout, depression, nurses’ well-being, work motivation, work engagement, job satisfaction and performance [57–59]. Additionally, Al-Bsheish et al. conducted serial mediation analyses and showed that psychological factors exerted indirect effects on safety compliance through a multiple-mediator model [60]. A literature review [13] has also shown that shift work alters psychophysical homeostasis, leading to a decrease in performance. Rotating-shift nurses face persistent high shift work demands, which may exceed their resource capacity, leading to depletion of physical, emotional, and cognitive resources. Such resource depletion may negatively affect safety motivation, behavior and work performance.

Moreover, the sleep quality and depressive symptoms of rotating-shift nurses could reflect the balance between shift work demands and available resources. When this balance is disrupted and resources are depleted, their motivation for safety behaviors, job performance, and even the stability of the healthcare system could be adversely affected. Therefore, hospital administrators and policymakers should urgently focus on optimizing job design, promoting a balance between shift work demands and resources, as well as developing rotating-shift nurses’ sleep and psychological health promotion strategies. However, this study focused only on depressive symptoms, and future research should further distinguish and examine the effects of other psychological factors, such as stress, burnout and resilience, on the safety behavior of rotating-shift nurses.

In this study, we further conducted a network analysis to examine the interrelationships between various factors in the sleep quality-depressive symptoms-safety behaviors network among rotating-shift nurses. In the items’ network, depressive symptoms showed stronger connections (ρₚ range: -0.25 to -0.17) with safety behavior level compared to sleep quality (ρₚ range: -0.17 to -0.02). Our findings are also similar to a network of sleep quality-anxiety-depression-burnout symptoms in the general population, which identified depressed mood as the most central symptom [61]. We also found depression symptoms D_3 (*Insomnia, disturbed sleep, or excessive sleep*) and PSQI_C3 (*Sleep duration*) were stable bridge symptoms in the network. Bridge symptoms highlight the most influential nodes in the network, connecting multiple symptoms. Interventions targeting these bridge symptoms can weaken the symptom network and disrupt pathological cascades [30, 32]. Therefore, “Insomnia, disturbed sleep, or excessive sleep” and “Sleep duration” represent potential targets for interventions in improving nurses’ sleep, psychological health and safety behavior. Practically, interventions could include optimizing shift schedules to allow sufficient rest, providing sleep hygiene education and cognitive behavioral therapy for insomnia, implementing brief breaks during shifts, and offering psychological support to reduce work-related stress. Such targeted interventions on bridge symptoms may effectively disrupt the pathological network and enhance overall safety behavior.

Moreover, we found that 70.6% of nodes in the network had clustering coefficients above 0.90, indicating a highly clustered structure. This suggests that most symptoms form tightly interconnected local clusters, which may facilitate symptom reinforcement within these clusters. Such high local clustering highlights the importance of targeting bridge or central symptoms to effectively disrupt the pathological network. However, further research is needed to explore these relationships and test interventions targeting these central symptoms.

This study has several limitations that should be acknowledged. First, the sample was drawn exclusively from healthcare institutions in Guangxi, China, which may limit the generalizability of the findings to rotating-shift nurses in other regions or organizational contexts. Second, several potentially confounders were not measured, including workload intensity, patient–nurse ratios, organizational safety culture, and medication use (e.g., sleep aids and antidepressants). These factors may influence sleep quality and depressive symptoms through increased occupational stress, and potentially affect the observed associations. Third, subgroup analyses were not conducted due to limited sample sizes in some groups, which would compromise the statistical power and stability of stratified analyses. In addition, the use of self-reported questionnaires may render the results susceptible to recall and reporting biases. Lastly, the cross-sectional design precludes causal inference and limits the examination of associations with objectively measured patient safety events. Future research should develop more objective and scientifically rigorous methods to assess healthcare professionals’ safety behaviors and better understand the potential mechanisms. Multicenter, longitudinal, or cohort studies incorporating a broader range of potential confounders and adequately powered subgroup analyses would be beneficial for clarifying the relationships between sleep quality, mental health, and safety behavior. They would also support more cautious generalization across regions and healthcare systems. Furthermore, employing multilevel frameworks in future studies could help investigate associations between safety behavior level and objectively measured patient safety outcomes.

## 5. Conclusion

Our study addresses an important gap in rotating-shift nurses’ sleep quality, depressive symptoms and safety behavior. The rotating-shift nurses exhibit relatively high levels of patient safety behavior despite experiencing significant sleep disturbances and depressive symptoms. The sleep quality of rotating-shift nurses influences their safety behaviors primarily through the mediating effect of depressive symptoms. Moreover, “Insomnia, disturbed sleep” of depressive symptoms, and “sleep duration” of sleep quality stand out as stable bridge symptoms in the sleep quality-depressive symptoms-safety behaviors network among rotating-shift nurses. Interventions targeting these bridge symptoms would benefit in improving nurse health and their patient safety behaviors.

## Supporting information

S1 Data The minimal dataset used for all analyses in the present study. The dataset includes variables required to replicate the reported findings, including sleep quality (PSQI Dimensions), depressive symptoms (PHQ-9 items), safety behavior levels (NSBQ items), and relevant demographic characteristics.
(XLSX)

S1 Table Direct, indirect (via PHQ-9 score), and total effects of sleep quality and covariates on safety behavior level in the fully adjusted mediation model. (XLSX)

S2 Table Spearman Correlation Coefficients (controlling for covariates) and 95% CI for all node pairs in the sleep quality-depressive symptoms-safety behaviors network. (XLSX)

S3 Table The node centrality metrics of each node in sleep quality- depressive symptoms-safety behaviors network. (XLSX)
